# Cu_2_O-Ag Tandem Catalysts for Selective Electrochemical Reduction of CO_2_ to C_2_ Products

**DOI:** 10.3390/molecules26082175

**Published:** 2021-04-09

**Authors:** Di Niu, Cong Wei, Zheng Lu, Yanyan Fang, Bo Liu, Da Sun, Xiaobin Hao, Hongge Pan, Gongming Wang

**Affiliations:** 1 Department of Applied Chemistry, University of Science and Technology of China, Hefei 230026, China; niudi@mail.ustc.edu.cn (D.N.); weicong@mail.ustc.edu.cn (C.W.); luzh2018@mail.ustc.edu.cn (Z.L.); fr1478@mail.ustc.edu.cn (Y.F.); liubo123@mail.ustc.edu.cn (B.L.); sunda727@mail.ustc.edu.cn (D.S.); 2 Institute of Science and Technology for New Energy, Xi’an Technological University, Xi’an 710021, China; honggepan@zju.edu.cn; 3 State Key Laboratory of Silicon Materials, School of Materials Science and Engineering, Zhejiang University, Hangzhou 310027, China

**Keywords:** carbon dioxide reduction, cuprous oxide, silver, tandem catalyst, C_2_ products

## Abstract

The electrochemical carbon dioxide reduction reaction (CO_2_RR) to C_2_ chemicals has received great attention. Here, we report the cuprous oxide (Cu_2_O) nanocubes cooperated with silver (Ag) nanoparticles via the replacement reaction for a synergetic CO_2_RR. The Cu_2_O-Ag tandem catalyst exhibits an impressive Faradaic efficiency (FE) of 72.85% for C_2_ products with a partial current density of 243.32 mA·cm^−2^. The electrochemical experiments and density functional theory (DFT) calculations reveal that the introduction of Ag improves the intermediate CO concentration on the catalyst surface and meanwhile reduces the C-C coupling reaction barrier energy, which is favorable for the synthesis of C_2_ products.

## 1. Introduction

Fossil fuels have been increasingly consumed since the Industrial Revolution, while substantial greenhouse gas CO_2_ is inevitably emitted into the atmosphere as a byproduct, along with the severe climate change [1,2,3]. Nowadays, reducing the emission of CO_2_ and recycling CO_2_ to value-added products is becoming a significant challenge that government has to face [4,5,6]. It is urgent to search for a clean and efficient way to convert CO_2_ to form an artificial carbon recycling [7,8]. With the rapid development of the electrochemistry and electrochemical catalysis, the electrochemical CO_2_ reduction reaction (CO_2_RR) to highly valuable C_2_ chemicals and fuels powered by renewable energy sources [9,10] represents one of the most environmentally friendly and sustainable strategies [11,12]. However, the catalytic process involved multiple electrons transfer generally suffers from low selectivity and high overpotentials [13,14,15,16]. Therefore, developing the electrocatalysts with the high selectivity and activity has been the focus of research.

Cu-based catalysts are the most-commonly used catalysts for CO_2_ reduction to C_2_ products such as ethylene, ethanol and acetate, because of its unique catalytic capability stemming from the electronic structures of copper [16,17,18,19]. The regulation by means of the morphology [20,21,22], composition [23,24,25,26] and the chemical state [27,28,29] has been applied to improve the performance of Cu-based catalysts. It has been demonstrated that cuprous oxide (Cu_2_O) plays a critical role in improving the selectivity toward C_2_ products [30,31,32,33,34]. For example, in 2012, Li et al. synthesized the modified Cu electrodes by reducing Cu_2_O films [30]. The thin Cu_2_O layers in the electrodes exhibited higher CO_2_ reduction activity than pure Cu metal. Later, Kas et al. prepared Cu_2_O derived copper nanoparticles and they found the selectivity of ethylene and ethane largely depended on the parent Cu_2_O film thickness [31]. The oxidized Cu catalysts prepared by Mistry et al. showed a 60% Faradaic efficiency (FE) towards ethylene [32]. Operando characterization and experimental results show that the presence of Cu^+^ is key to lowering the onset potential and enhancing ethylene selectivity. In 2017, Xiao et al. used density functional theory (DFT) calculations to research on the advantages of Cu_2_O-derived electrodes [34]. The results show that Cu^+^ has the ability to promote CO_2_ activation and the Cu^+^ and Cu^0^ cooperatively improves the kinetics and thermodynamics of both CO_2_ activation and CO dimerization, thereby boosting the efficiency and selectivity of CO_2_RR. While Cu_2_O exhibits the enhanced performance in the activity and selectivity for CO_2_ reduction to C_2_ products, it still cannot meet the decent selectivity and activity for the industry.

Since CO is an important reaction intermediate for the C-C coupling reaction in CO_2_RR to C_2_ products, increasing the near-surface CO concentration (and consequently CO surface coverage) is a key factor that can enhance the selectivity towards C_2_ products [35,36,37]. Considering that Ag owns the ability to reduce CO_2_ to produce CO [38,39,40], cooperating Cu_2_O with Ag may be an effective strategy to improve the selectivity of C_2_ products. Previous studies have reported a lot of Cu-Ag catalysts [41,42,43,44,45], and recently a Cu-Ag tandem catalyst was synthesized by Chen et al., which resulted in a four-fold enhancement of C_2_ products formation on Cu [43]. The Ag-incorporated biphasic Cu_2_O-Cu catalysts synthesized by Lee et al. reached an FE for C_2_ products of 49% [44]. Later, improved FEs for ethylene and ethanol of nearly 55% and 26% were achieved using nanoporous Cu-Ag alloys [45]. While the FE of C_2_ products have been improved, the activity remains low and the current densities are not high enough to meet the commercial purpose. Moreover, the reaction mechanism for converting CO_2_ to C_2_ products on the Cu_2_O-Ag catalyst also needs to be further revealed.

In this work, we prepared Ag modified Cu_2_O (Cu_2_O-Ag) tandem catalysts with a size of ~50 nm by a replacement reaction for CO_2_RR. The electrochemical studies reveal that introducing Ag into Cu_2_O can substantially boost the generation of CO and improve the FE of C_2_ products to 72.85% at −1.18 V (vs. RHE). The maximum FE of acetate reaches 15.03% at −1.18 V (vs. RHE) on the Cu_2_O-Ag catalyst, which is three times higher than that of the Cu_2_O catalyst at the same potential. The experiments results and DFT calculations show that high coverage of CO on the catalyst surface reduces the C-C coupling barrier energy, which is helpful in the synthesis of the C_2_ products.

## 2. Results and Discussion

**Figure 1:**
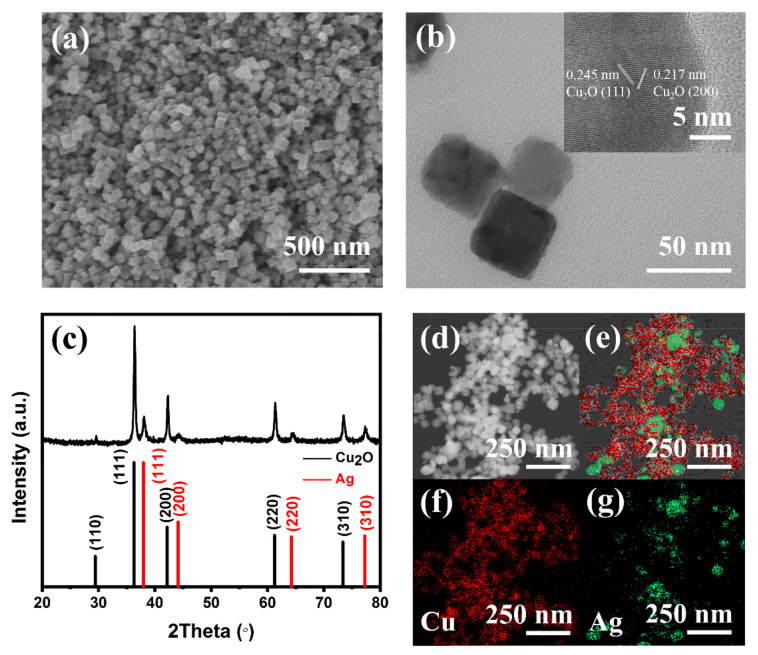
(**a**) SEM image of Cu_2_O-Ag nanocubes. (**b**) TEM and (inset) HRTEM images of Cu_2_O-Ag nanocubes. (**c**) XRD pattern of Cu_2_O-Ag nanocubes. (**d**–**g**) HRTEM-EDS elemental mapping of Cu_2_O-Ag nanocubes, identifying homogeneously distributed (**f**) Cu and (**g**) Ag.

Figure 1a shows the scanning electron microscopy (SEM) image of the prepared Cu_2_O-Ag catalysts, which displays uniform cubic morphology. The average size of the densely distributed nanocubes is ~50 nm. Transmission electron microscopy (TEM) image in Figure 1b shows that the Cu_2_O-Ag has the regular shapes with the average edge length of 50 ± 8 nm (Figure S1a,b). The morphologies and structures of Cu_2_O nanocubes (Figures S1c,d and S2a,b) are similar to that of the Cu_2_O-Ag nanocubes, suggesting that the Cu_2_O-Ag nanocubes preserve the original morphologies and structures of Cu_2_O. Moreover, the lattice fringes of Cu_2_O-Ag and Cu_2_O were observed by high–resolution transmission electron microscopy (HRTEM). The interplanar spacing of 0.245 and 0.217 nm of Cu_2_O-Ag is assigned to the (111) and (200) plane of Cu_2_O (Figure 1b), respectively, which are also observed in the Cu_2_O nanocubes (Figure S2c), further confirming that the phase of Cu_2_O is preserved after adding Ag. Figure 1c and S2d exhibit the X-ray diffraction (XRD) patterns for Cu_2_O-Ag and Cu_2_O, respectively. Both of these catalysts exhibit similar profiles, where the diffraction peaks at 2θ = 29.6°, 36.4°, 42.3°, 61.4° and 73.5° belong to the planes of Cu_2_O(110), (111), (200), (220) and (210) (JCPDS 05-0667), respectively. After introducing Ag, the new peaks at 2θ = 38.0°, 44.3°, 64.4° and 77.3° corresponding to planes of Ag(111), (200), (220) and (310) (JCPDS 04-0783) appear, indicating the formation of Ag sites in the Cu_2_O-Ag catalysts. Energy dispersive X-ray spectroscopy (EDS) elemental mapping was further used to prove the existence and distribution of Ag. SEM-EDS elemental mapping images (Figure S3) confirm the existence of Cu and Ag elements, which are homogeneously distributed among the catalysts. HRTEM-EDS (Figure 1d–g) elemental mapping images show that Ag particles exist among Cu_2_O nanocubes and the particle size of Ag is smaller than that of Cu_2_O nanocubes. These results clearly reveal that the Cu_2_O-Ag catalyst has been prepared with separated Cu_2_O nanocubes and Ag nanoparticles.

**Figure 2:**
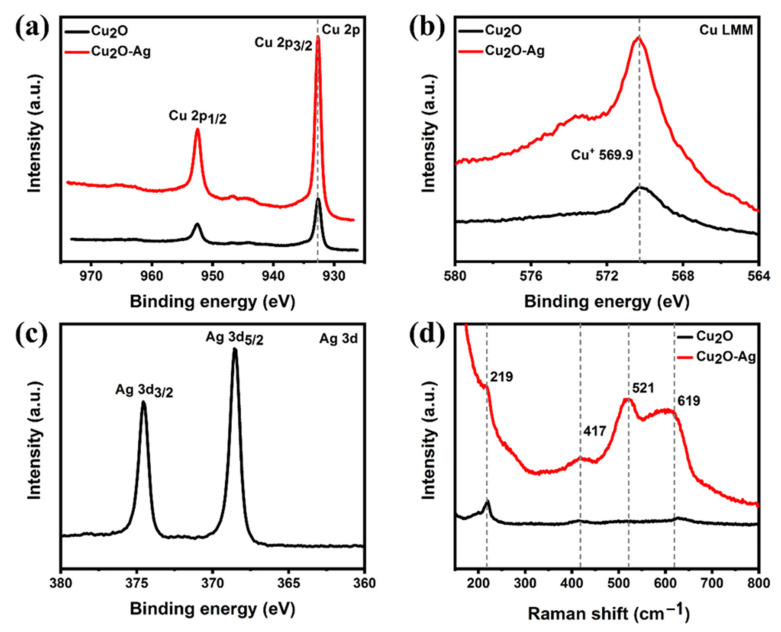
(**a**) XPS Cu 2p spectra. (**b**) Auger spectra of Cu LMM. And (**c**) XPS Ag 3d spectra of Cu_2_O-Ag nanocubes. (**d**) Raman spectra of Cu_2_O-Ag and Cu_2_O nanocubes, respectively.

The X-ray photon spectroscopy (XPS) spectrum was further conducted to probe the chemical and composition states of Cu_2_O-Ag and Cu_2_O catalysts. Figure 2a shows the Cu 2p spectra of Cu_2_O-Ag and Cu_2_O. Both catalysts display one pair of spin-orbit doublet peaks of Cu 2p, which can be assigned to Cu 2p_3/2_ and Cu 2p_1/2_, respectively. The peaks located at 932.7 and 952.5 eV are attributed to the Cu 2p_3/2_ and Cu 2p_1/2_ of the Cu_2_O (Cu^+^) or Cu (Cu^0^), respectively [46]. After introducing Ag, the binding energies of Cu 2p_3/2_ and Cu 2p_1/2_ only exhibit a little shift to the lower energy region. It is difficult to distinguish the Cu^+^ and Cu^0^ chemical state through XPS because the difference of the binding energies between Cu^+^ and Cu^0^ is only 0.1 eV [46]. Auger electron spectroscopy (AES) was used to further verify the chemical state of Cu. Figure 2b clearly shows the signal of Cu^+^ state at 569.9 eV for both samples, which is different from the metallic Cu^0^ state at 568.0 eV in the reported literature [47]. The Ag 3d_3/5_ and 3d_3/2_ peaks of the Cu_2_O-Ag are located at 368.6 and 374.6 eV (Figure 2c), which are consistent with the literature values of metallic Ag [48]. Furthermore, Raman spectra were also used to confirm the oxide state of Cu. In Figure 2d, both samples exhibit Raman peaks at around 219, 417, 521 and 619 cm^−1^, which correspond to the 2Γ^−^_12_, 4Γ^−^_12_, Γ^+^_25_ and Γ^−^_12_ + Γ^+^_25_ phonon modes of Cu_2_O [49], respectively. These results are in good agreement with the previous XRD and HRTEM measurements, confirming the formation of the Cu_2_O sample and the Cu_2_O-Ag tandem catalyst.

**Figure 3:**
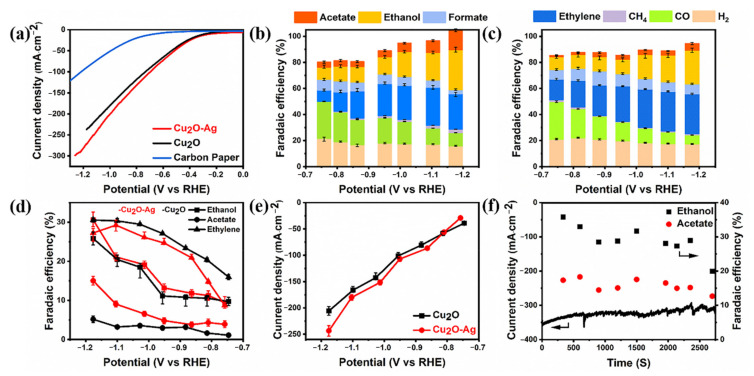
(**a**) LSV curves on the Cu_2_O-Ag and Cu_2_O nanocubes in 1 M KOH electrolytes. Blue curves obtained from the blank GDL in 1 M KOH electrolytes. (**b**) Faradaic efficiencies of all CO_2_RR products on the Cu_2_O-Ag nanocubes. (**c**) Faradaic efficiencies of all CO_2_RR products on the Cu_2_O nanocubes. (**d**) Faradaic efficiencies of ethylene, acetate and ethanol of Cu_2_O-Ag and Cu_2_O nanocubes at different applied potentials. (**e**) Partial current densities of C_2_ products of Cu_2_O-Ag and Cu_2_O nanocubes at different applied potentials. (**f**) Stability test in 1M KOH electrolytes at −1.18 V versus RHE.

The selectivity and activity of CO_2_RR were tested in a commercial flow-cell using gas diffusion electrode (GDE) at constant applied electrode potentials in aqueous 1 M KOH electrolyte. A cation-exchange membrane (Nafion N115, DuPont) separates the cathode compartment from the anode compartment. The catalyst powder dispersed in methanol are applied on a hydrophobic carbon paper using as the gas diffusion layer (GDL) and the GDL is used as the GDE. CO_2_ is flowing through the cell at the backside of the GDL. The ambient CO_2_ pressure minimizes mass transport limitations and enables high currents compared to H-type electrolytic cell test [50]. As shown in Figure 3a, the linear sweep voltammetry (LSV) curves were obtained at 10 mV·s^−1^ in 1 M KOH electrolyte on the Cu_2_O-Ag and Cu_2_O catalyst, respectively. Both catalysts show the sharp reduction peaks for CO_2_ reduction and higher current densities compared to blank carbon paper. As the potential decreases from −0.40 to −1.15 V (vs. RHE), the total current densities of Cu_2_O-Ag and Cu_2_O increase from 25.31 mA·cm^−2^ to 260.80 mA·cm^−2^ and from 22.16 to 229.40 mA·cm^−2^, respectively. Therefore, the Cu_2_O-Ag and Cu_2_O catalysts show the similar trends in total current densities, but the current densities of Cu_2_O-Ag are higher than that of Cu_2_O.

The FE values of CO_2_ reduction products on the Cu_2_O-Ag and Cu_2_O catalysts are shown in Figure 3b,c, respectively. For Cu_2_O-Ag catalysts, CO and H_2_ are the dominant products with total 50.01 ~ 37.57% FE at low overpotentials from −0.76 V to −0.95 V (vs. RHE). However, the FEs of CO and H_2_ decrease to 10.15% and 15.92% as the potential decreases to −1.18 V (vs. RHE). Importantly, the FE of all C_2_ products increases from 21.74% at −0.76 V to 72.85% at −1.18 V (vs. RHE). Ethylene and ethanol are the primary C_2_ products with the FEs of 27.23% and 30.60% at −1.18 V (vs. RHE) (Figure 3d), respectively. The FE of acetate also increases from 3.90% to 15.02% as the potential decreases from −0.76 V to −1.18 V (vs. RHE) (Figure 3d). Compared with the Cu_2_O-Ag catalyst, the Cu_2_O catalyst shows slightly lower FE of CO and higher FE of H_2_, ranging from 28.81% and 21.04% at −0.75 V to 6.86% and 17.43% at −1.18 V (vs. RHE), respectively, while the FEs of ethylene, ethanol and acetate increase from 15.95%, 9.74% and 1.01% at −0.75 V to 30.55%, 25.84% and 5.18% at −1.18 V (vs. RHE) (Figure 3d), respectively. While the FEs of all C_2_ products increases from 26.70% at −0.75 V to 61.57% at −1.18 V (vs. RHE), which shows a similar trend to the Cu_2_O-Ag catalyst, the Cu_2_O-Ag catalyst has the higher FE of C_2_ products and lower FE of H_2_.

Furthermore, as shown in Figure 3d,e, the Cu_2_O-Ag catalyst reaches a maximum C_2_ products FE of 72.85% at −1.18 V (vs. RHE) with the partial current density of 243.32 mA·cm^−2^, involving 27.23% ethylene, 30.60% ethanol and 15.02% acetate. Compared to Cu_2_O-Ag, the total C_2_ products FE of Cu_2_O is 61.57% with the partial current density of 205.64 mA·cm^−2^, involving 30.55% ethylene, 25.84% ethanol and only 5.18% acetate. Obviously, the FE of acetate on the Cu_2_O-Ag is about three times higher than that on the Cu_2_O, suggesting a more prominent ability of Cu_2_O-Ag than Cu_2_O for CO_2_ reduction to acetate.

The stability of the Cu_2_O-Ag catalyst was tested at −1.18 V (vs. RHE) (Figure 3f). The catalyst shows a good performance without the decay of the current density and C_2_ product FEs in 40 min. The FEs of ethanol and acetate remain over 30% and 15% for over 2400 s with a total current density of over 300 mA cm^−2^. As shown in Figures S4 and S5, the catalyst morphology and crystal facet were changed at 5 min after CO_2_RR and continuously changed with the reaction progress, while the performance is basically unchanged. Hence, the change in catalyst morphology and crystal facet might not be the main reason of performance degradation and the decrease of C_2_ products FE and the current density may be due to the loss of hydrophobicity over the GDLs and the salinization of electrolyte [51,52,53,54]. From the above results, it can be concluded that the Cu_2_O-Ag is more active and selective for CO_2_RR to C_2_ products than the Cu_2_O catalyst, especially producing acetate.

After the CO_2_RR test, SEM and XRD were used to probe the change of morphologies and compositions of both catalysts. The SEM images show that the morphologies of Cu_2_O in both Cu_2_O-Ag and Cu_2_O catalyst are changed to irregular particles after the catalytic reaction (Figure S6). Small nanocubes agglomerate together to form larger and irregular shape particles under the negative potential conditions with a particle size of over 100 nm and each particle can be clearly distinguished from others. At the negative potentials, the Cu_2_O is easily reduced [55]. As shown in Figure S7, the new peaks at 43.2° and 50.3° corresponding to Cu (JCPDS 04-0836) in XRD patterns are showed, indicating that most of Cu_2_O is reduced to Cu after CO_2_RR. However, Ag peaks and Cu_2_O peaks are still existed in XRD patterns, which means that Ag and a small amount of Cu_2_O are preserved after CO_2_RR. EDS elemental mapping (Figure S8a–c) also confirms that Ag still exists and is homogeneously distributed on the GDL. The content of Ag investigated by inductively coupled plasma-atomic emission spectrometry (ICP-AES) decreases from 9.72% to 7.39% after CO_2_RR (Table S1). The reason for the decreased Ag content might be that it is flushed away by electrolytes. XPS results of Cu_2_O-Ag after CO_2_RR in Figure S9 show the characteristic metallic Ag peaks and mixed Cu^0^ + Cu^+^ peaks, confirming the existence of Ag and reduction of Cu_2_O to Cu, and the XPS spectrum of Cu_2_O after CO_2_RR also shows the mixed Cu^0^ + Cu^+^ peaks (Figure S10) [56,57,58].

**Figure 4:**
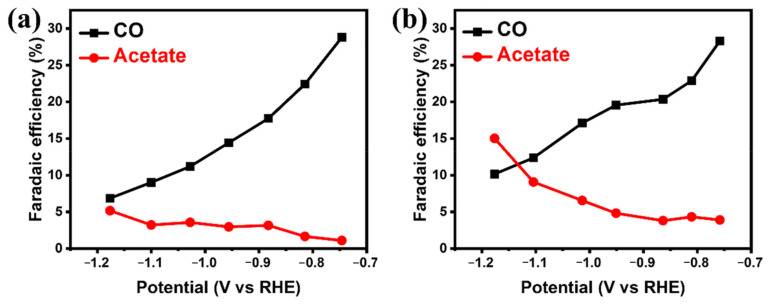
CO and acetate Faradaic efficiencies on (**a**) Cu_2_O and (**b**) Cu_2_O-Ag nanocubes at different applied potentials.

In order to investigate the mechanism of higher FEs of C_2_ products, the following experiments are further studied. As shown in Figure 3, the introduction of Ag promotes producing C_2_ products. The FE of acetate increases as the mass ratios of Ag increase (Figure S11). When Ag is introduced into Cu_2_O, it acts as the CO active site, creating a high surface concentration of CO, which benefits CO dimerization and the synthesis of C_2_ products [59]. The produced CO can be transferred neighboring Cu, along with C-C coupling reaction to produce C_2_ products, which results in the highly selective CO dimerization. Moreover, a higher CO concentration also benefits from increasing the reaction rate of the C-C coupling step and FEs of C_2_ products. As shown in Figure 4a,b, the Cu_2_O-Ag catalyst produces more CO at high overpotentials than Cu_2_O, corresponding to the increases of acetate FE. At the potential of −1.18 V (vs. RHE), the FE of CO for Cu_2_O-Ag catalyst is 10.15%, corresponding to 15.02% acetate. In contrast, the Cu_2_O catalyst produce 6.86% FE of CO, corresponding to 5.18% FE of acetate. These experimental results show that the FE of CO is related to that of acetate and a high CO concentration may be helpful to the synthesis of C_2_ products, which is further revealed by the following DFT calculations.

**Figure 5:**
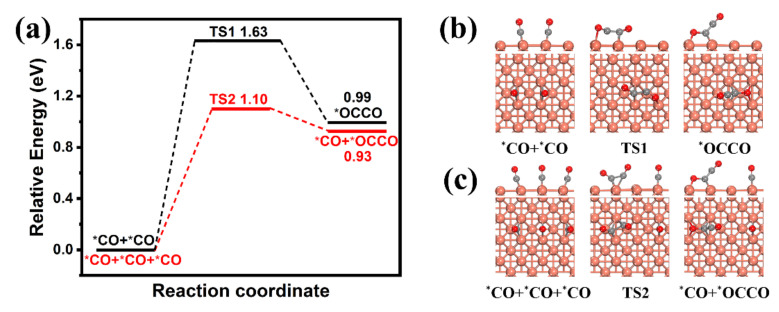
(**a**) Reaction barrier energies of C-C coupling step at the low and high CO concentrations; the corresponding configurations of initial state, transition state and final state on the CO coverage of (**b**) 2/9 ML and (**c**) 3/9 ML. Light red, copper; grey, carbon; red, oxygen; TS, transition state; * represents adsorbates).

Cu_2_O and Ag are regarded as an important active site for converting CO_2_ to CO [34,60]. Therefore, in our study, not only Cu_2_O, but also Ag, can act as active sites producing CO from CO_2_. More CO can react with each other on Cu surface to form C-C bond and further C_2_ product. DFT calculations were applied to investigate the effect of CO coverage on the C-C coupling step over the Cu catalyst, which is the key fundamental step for the synthesis of C_2_ products. Considering the reduction of Cu_2_O to Cu during the CO_2_ reduction process, the Cu surface, with the (100) plane having the *p*(3 × 3) size, was constructed to model the catalyst [61]. The most stable adsorption site of CO on Cu(100) surface is firstly explored. As shown in Figure S12, CO is preferred to adsorb at the bridge site, which accords well with the previous study [62]. In order to model the effect of the high CO concentration on Cu producing and spilling from Ag, we compared the reaction barrier energies of the C-C coupling step at the CO coverage of 3/9 ML with 2/9 ML, which represents the high and low CO concentration on the catalyst surface, respectively. As shown in Figure 5, there is the lower C-C coupling barrier energy (1.10 eV) on 3/9 ML than that (1.63 eV) on 2/9 ML, which indicates that the high coverage of CO promotes the C-C coupling step and the C_2_ products. However, the barrier energy of C-C coupling reaction on the *p*(2 × 2) Cu_2_O(100) surface (3.03 eV) at the CO coverage of 1/2 ML is much higher than those on the Cu surface at different coverages (1.63 and 1.10 eV) (Figure S13), which suggests that Cu is more favorable for the C-C coupling reaction than Cu_2_O.

## 3. Materials and Methods

### 3.1. Materials

Copper sulfate (CuSO_4_·5H_2_O, 99%), sodium hydroxide (NaOH, 99%), ascorbic acid (C_6_H_8_O_6_, 99.7%) and silver nitrate (AgNO_3_, 99.95%) were purchased from Shanghai Sinopharm Chemical Reagent Co., Ltd. (Shanghai, China) and were used without purification.

### 3.2. Synthesis of Cu_2_O Nanocubes

The Cu_2_O nanocubes were synthesized by a previously reported method [63]. In a typical synthesis, 0.3 g CuSO_4_·5H_2_O was dissolved in 400 mL deionized water (DIW) and stirred for 30 min at 20 °C. Then, 1 mL 4.8 M NaOH solution was added slowly. After 5 min, 1 mL 1.2 M ascorbic acid was injected. The solution was further stirred for 30 min and the solution color was turned from blue to orange. The sample was washed with DIW and ethanol at least 3 times, respectively. Then, it was dried at 60 °C in a vacuum oven overnight.

### 3.3. Synthesis of Cu_2_O-Ag Nanocubes

The Cu_2_O nanocubes were synthesized following the above-mentioned steps. After the Cu_2_O nanocubes solution was prepared, a certain amount of silver nitrate solution (35 mL, 2 mmol) was injected. The solution color was turned from orange to black immediately and then stirred for 5 min. The sample was washed with DIW and ethanol at least 3 times. Then, it was dried at 60 °C in a vacuum oven overnight.

### 3.4. Material Characterizations

The prepared samples were characterized by the following techniques. X-ray diffraction (XRD) measurements were performed on a Philips X’Pert Pro Super diffractometer (Philips, Almelo, The Netherlands) using Cu Kα radiation (λ  =  1.54178 Å). The morphological structures were collected using a JEOL-2010-JSM-6700F scanning electron microscopy (SEM) (JEOL, Tokyo, Japan) and Hitachi H7650 transmission electron microscopy (TEM) (Hitachi, Tokyo, Japan). Energy dispersive X-ray spectroscopy (EDS) mapping and high–resolution transmission electron microscopy (HRTEM) images were acquired on Talos F200X (FEI, Hillsboro, OR, USA) and JEMARM 200F microscope (JEOL, Tokyo, Japan). Raman spectra were recorded on a Renishaw RM 3000 Micro-Raman system (Renishaw, Gloucestershire, UK) with a 532 nm excitation laser. X-ray photon spectroscopy (XPS) and Auger electron spectroscopy (AES) were performed at Thermo Scientific ESCALAB 250Xi X-ray Photoelectron Spectrometer (Thermo, Waltham, MA, USA).

### 3.5. Electrochemical Measurements

All the electrochemical measurements were performed in a three-electrode system on CHI 760E electrochemical workstation (Chenhua, Shanghai, China). The potentials were measured against an Ag/AgCl reference electrode and converted to the reversible hydrogen electrode (RHE) using the equation:

*E* (vs. RHE) = *E* (vs. Ag/AgCl) + 0.197 + (0.059 × pH) (1)

A gas diffusion layer (GDL) was used as the catalyst support. We dispersed 5 mg catalyst powder in 1 mL methanol and 40 μL 5 wt% Nafion. The ink was sonicated for 30 min and dripped on the top of GDL (1 cm^2^). Then, the GDL was dried in a vacuum oven at 60 °C for 4 h. The GDL was weighed before and after catalyst deposition and loading contents were 1 mg cm^−2^.

The electrolysis investigations were carried out in a flow cell purchased from Gaossunion (Gaossunion, Wuhan, China). The cathode (GDL) and anode (nickel foam) compartments were separated by an anion exchange membrane. The CO_2_ gas chamber was located behind the cathode chamber and separated from the catholyte with a GDL. The CO_2_ flow rates were set at 20 mL min^−1^. Electrolytes were circulated through cathode and anode through the cell at 10 mL min^−1^ using peristaltic pump. 1 M KOH (25 mL) was used as electrolyte solutions. All electrolyte solutions were prepared with deionized water (18.2 MΩ cm).

### 3.6. Product Analysis

Gas and liquid products were quantified by gas chromatography (GC) (GC9560, Awa, Shanghai, China) and nuclear magnetic resonance (NMR) spectroscopy (AVANCE 400, Bruker, Fällanden, Switzerland), respectively. Faradaic efficiencies (FE) of gas and liquid products were calculated using the following equation:

FE (%) = *eFn*/*Q* × 100% = *eFn*/*It* × 100% (2)

where *e* is the number of electrons transferred, *F* is the Faraday constant, *n* is the amount of product in moles, *Q* is the charge, *I* is the current and *t* is the electrolysis time.

### 3.7. Theoretical Calculation

Density functional theory (DFT) calculation, performed by the Vienna Ab-initio Simulation Package (VASP 5.3) code (University of Vienna, Vienna, Austria), was used in this study [64,65,66]. The electron exchange-correlation potential was conducted by the Perdew-Burke-Ernzerhof (PBE) functional of generalized gradient approximation (GGA) [67,68]. The kinetic energy cut-off energy was set to 520 eV for the plane-wave basis set and the DFT dispersion correction (DFT-D3) [69] method was used to treat the van der Waals interactions. Brillouin zone integration was sampled with the 9 × 9 × 9 and 3 × 3 × 1 Monkhorst-Pack mesh k-point for bulk and surface calculations, respectively. The convergence of geometric optimization was checked with the forces less than 0.01 eV·Å^−1^ and the energy difference less than 1 × 10^−5^ eV. The climbing-image nudged elastic band method (CI-NEB) [70,71] was employed to obtain the approximate transition statessaddle point, followed by the dimer method [72], which was carried out to further optimize the transition states with the convergence criteria of the force acting on the atom less than 0.05 eV Å. Meanwhile, the transition state was verified using the single imaginary frequency. In addition, a U-J value of 3 eV was used for DFT+U correction on the Cu_2_O(100) surface. The adsorption energy (*E*_ads_), activation energy (*E*_a_) and reaction energy (Δ*E*) were obtained by the formulas:

*E*_ads_ = *E*_CO/substrate_ − *E*_CO_ − *E*_substrate_ (3)

*E*_a_ = *E*_TS_ − *E*_IS_ (4)

Δ*E* = *E*_FS_ − *E*_IS_ (5)

where *E*_ads_ is the adsorption energy of *CO on the surface, *E*_CO/substrate_ is the total energy of substrate and CO, *E*_CO_ is the energy of CO, *E*_substrate_ is the energy of substrate; *E*_a_ and Δ*E* are the barrier and the reaction energy, respectively; *E*_IS_, *E*_TS_ and *E*_FS_ are the energy of initial state, transition state and final state, respectively.

## 4. Conclusions

In summary, we prepared a Cu_2_O-Ag tandem catalyst for CO_2_ electrochemical reduction reaction through a facile synthetic method. The tandem catalyst made up of Cu_2_O and Ag nanoparticles improves the selectivity and activity of CO_2_ reduction toward C_2_ products compared to Cu_2_O catalyst. At the potential of −1.18 V (vs. RHE), the Cu_2_O-Ag catalyst shows a maximum C_2_ products FE of 72.85% with a partial current density of −243.32 mA·cm^−2^. It has an FE of 15.02% towards acetate, which is three times higher than that of Cu_2_O. Based on further experiments and DFT calculation, we found that increased CO concentration, produced on the Ag sites, plays an important role towards the production of acetate. The study shows a simple method to improve C_2_ production and provides deeper insights into designing the catalysts for CO_2_ electrochemical reduction.

## Data Availability

The data presented in this study are available in the article and supplementary material.

## Supplementary Materials

The following data are available online, Figure S1. (a) TEM image and (b) size distribution of Cu_2_O-Ag nanocubes; (c) TEM image and (d) size distribution of Cu_2_O nanocubes. Figure S2. (a) SEM image of Cu_2_O nanocubes; (b) TEM image of Cu_2_O nanocubes; (c) HRTEM image of Cu_2_O nanocubes; (d) XRD pattern of Cu_2_O nanocubes. Figure S3. (a–c) SEM-EDS elemental mapping of Cu_2_O-Ag nanocubes, showing the distribution of elemental (b) Cu and (c) Ag within the particles. Figure S4. The SEM images of the Cu_2_O-Ag during CO_2_RR. (a) 0 min, (b) 5 min, (c) 10 min, (d) 15 min, (e) 20 min, (f) 25 min, (g) 30 min, (h) 35 min, (i) 40 min. Scale bar = 500 nm. Figure S5. The XRD pattrens of the Cu_2_O-Ag during CO_2_RR. Figure S6. SEM images of (a) Cu_2_O-Ag nanocubes and (b) Cu_2_O nanocubes after CO_2_RR. Figure S7. XRD patterns of (a) Cu_2_O-Ag nanocubes and (b) Cu_2_O nanocube after CO_2_RR. Figure S8. (a–c) SEM-EDS elemental mapping of Cu_2_O-Ag nanocubes of elemental (b) Cu and (c) Ag after CO_2_RR. Table S1. Cu and Ag contents of Cu_2_O-Ag nanocubes before and after CO_2_RR. Figure S9. XPS spectra for (a) Cu and (b) Ag of Cu_2_O-Ag nanocubes after CO_2_RR. Figure S10. XPS spectra for Cu of Cu_2_O nanocubes after CO_2_RR. Figure S11. Faradaic efficiency of acetate of the different mass ratios of Ag in the Cu_2_O-Ag nanocubes. Figure S12. Adsorption energy and corresponding configuration of CO at three type sites on Cu(100) surface. Figure S13. (a) Reaction energy barrier diagram of the C-C coupling step on the Cu_2_O(100) surface with the (b)corresponding configurations of two ^*^CO forming an ^*^OCCO. Light red, copper; grey, carbon; red, oxygen; TS, transition state.

## Sample Availability

Samples of the compound are available from the authors.
