# Supplementary material for: Cu2O-Ag Tandem Catalysts for Selective Electrochemical Reduction of CO2 to C2 Products
Source: Molecules. 2021 Apr 9;26(8):2175. doi: 10.3390/molecules26082175 (PMC8070483; doi:10.3390/molecules26082175)
Supplement: Supplementary file 1 [file molecules-26-02175-s001.pdf]

molecules

Supporting Information

Cu_2O-Ag Tandem Catalysts for Selective Electrochemical Reduction of CO_2 to C_2 Products

Di Niu [1], Cong Wei [1], Zheng Lu [1], Yanyan Fang [1], Bo Liu [1], Da Sun [1], Xiaobin Hao [1,*], Hongge Pan [2,3] and Gongming Wang [1,*]

[1] Department of Applied Chemistry, University of Science and Technology of China, Hefei 230026, China; niudi@mail.ustc.edu.cn (D.N.); weicong@mail.ustc.edu.cn (C.W.); luzh2018@mail.ustc.edu.cn (Z.L.); fr1478@mail.ustc.edu.cn (Y.F.); liubo123@mail.ustc.edu.cn (B.L.); sunda727@mail.ustc.edu.cn (D.S.)
[2] Institute of Science and Technology for New Energy, Xi'an Technological University, Xi'an 710021, China; honggepan@zju.edu.cn (H.P.)
[3] School of Materials Science and Engineering, State Key Laboratory of Silicon Materials, Zhejiang University, Hangzhou 310027, China
* Correspondence: haoxb@ustc.edu.cn (X.H.); wanggm@ustc.edu.cn (G.W.); Tel: +86-551-63601051 (G.W.)

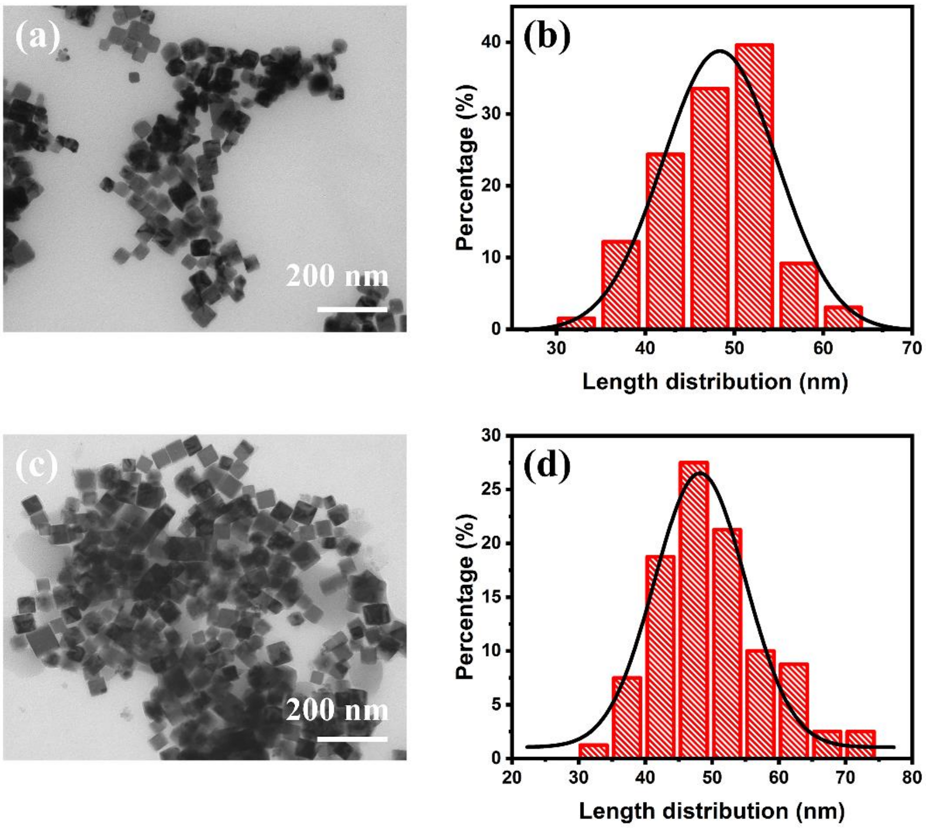

Figure S1. (**a**) TEM image and (**b**) size distribution of Cu_2O-Ag nanocubes. (**c**) TEM image and (**d**) size distribution of Cu_2O nanocubes.

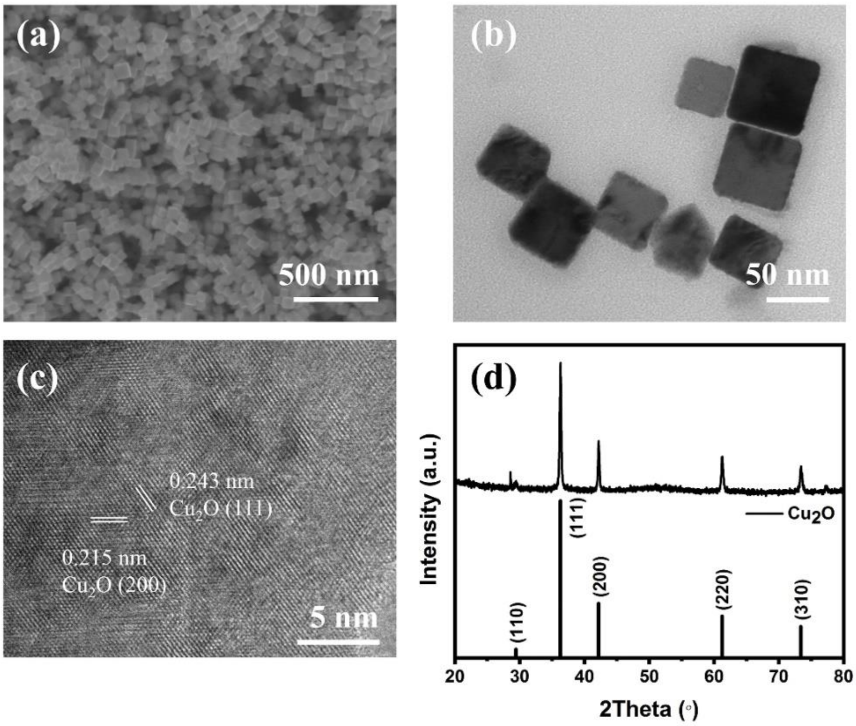

Figure S2. (**a**) SEM image of Cu_2O nanocubes. (**b**) TEM image of Cu_2O nanocubes. (**c**) HRTEM image of Cu_2O nanocubes. (**d**) XRD pattern of Cu_2O nanocubes.

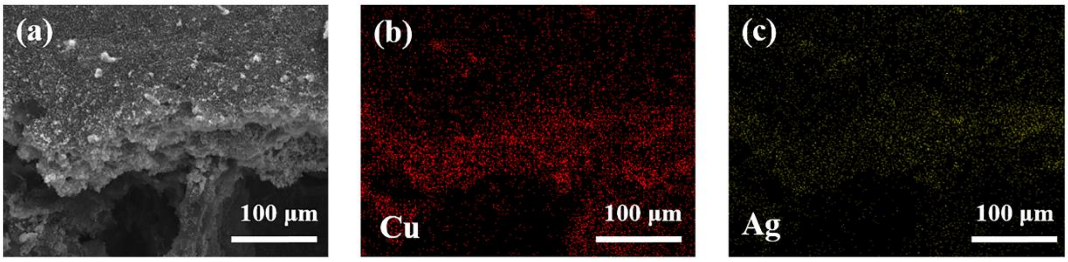

Figure S3. (**a**–**c**) SEM-EDS elemental mapping of Cu_2O-Ag nanocubes, showing the distribution of elemental (**b**) Cu and (**c**) Ag within the particles.

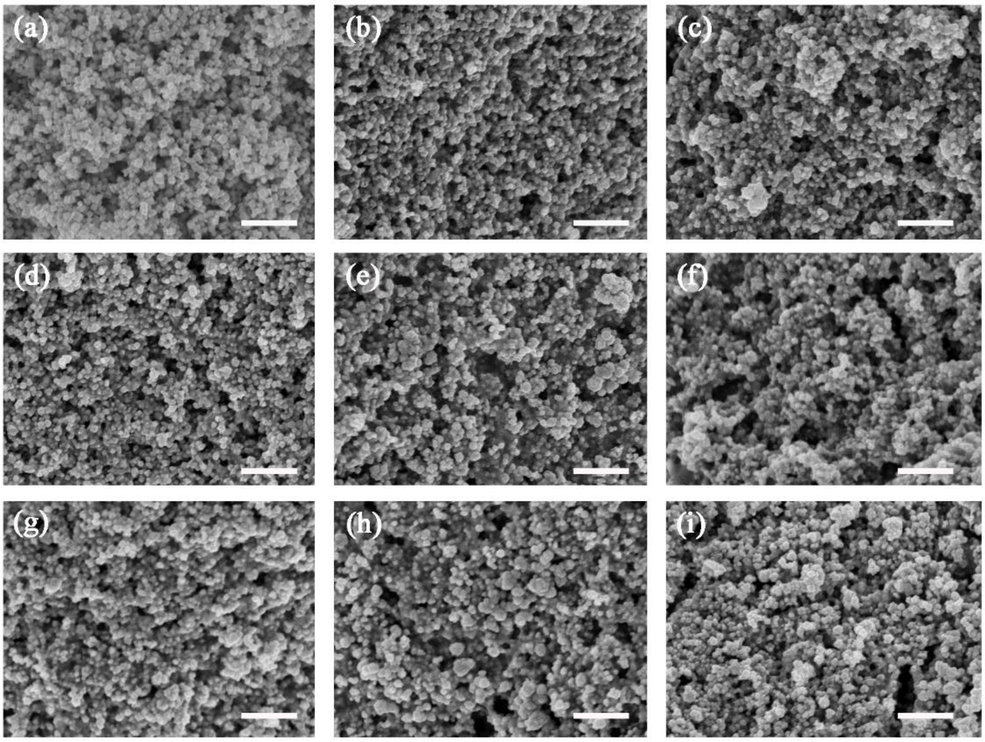

Figure S4. The SEM images of the Cu_2O-Ag during CO_2RR. (**a**) 0 min, (b) 5 min, (**c**) 10 min, (**d**) 15 min, (**e**) 20 min, (**f**) 25 min, (**g**) 30 min, (**h**) 35 min, (**i**) 40 min. Scale bar=500 nm.

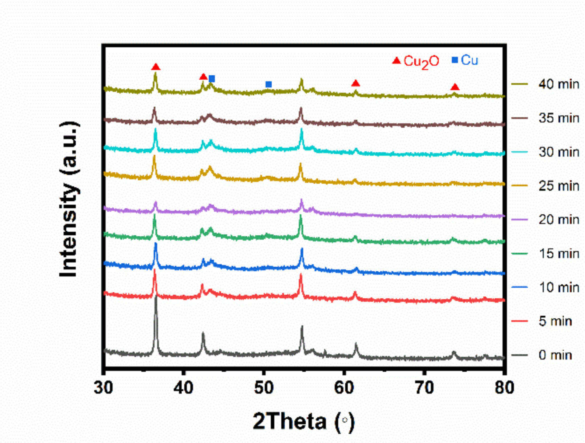

Figure S5. The XRD patterns of the Cu_2O-Ag during CO_2RR.

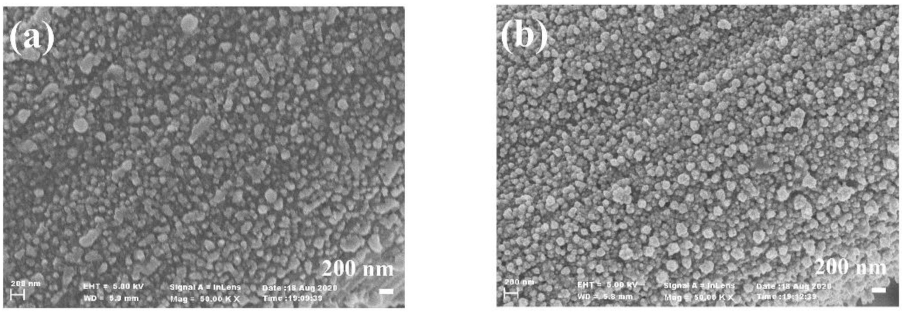

Figure S6. SEM images of (**a**) Cu_2O-Ag nanocubes and (**b**) Cu_2O nanocubes after CO_2RR.

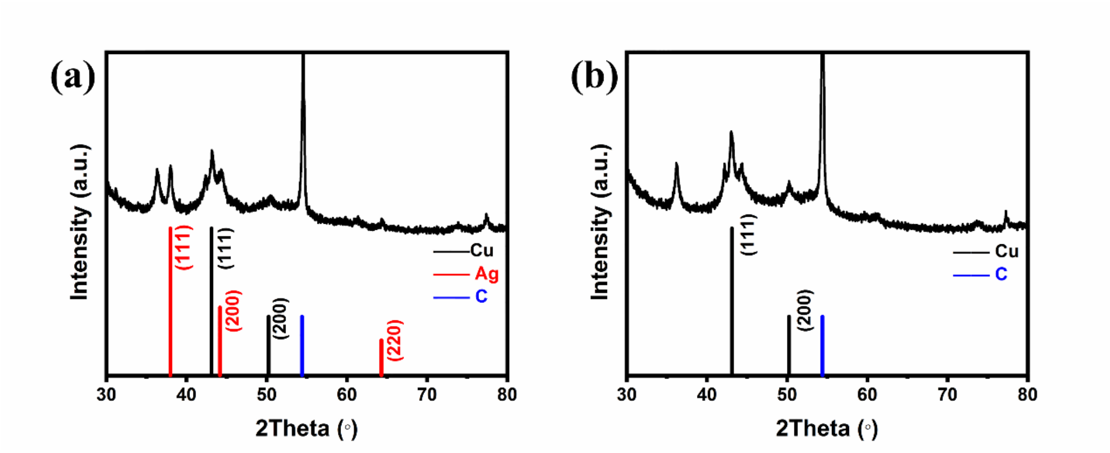

Figure S7. XRD patterns of (**a**) Cu_2O-Ag nanocubes and (**b**) Cu_2O nanocubes after CO_2RR.

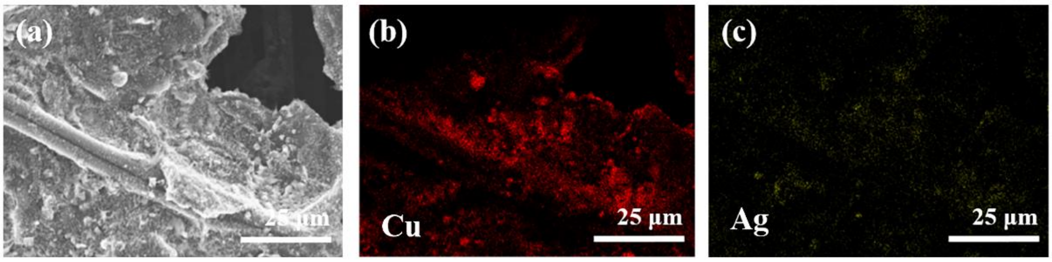

Figure S8. (**a**–**c**) SEM-EDS elemental mapping of Cu_2O-Ag nanocubes of (**b**) Cu and (**c**) Ag after CO_2RR.

Table S1. Cu and Ag contents of Cu_2O-Ag nanocubes before and after CO_2RR.

Element Content	Cu	Ag
Before CO_2RR	90.28%	9.72%
After CO_2RR	92.61%	7.39%

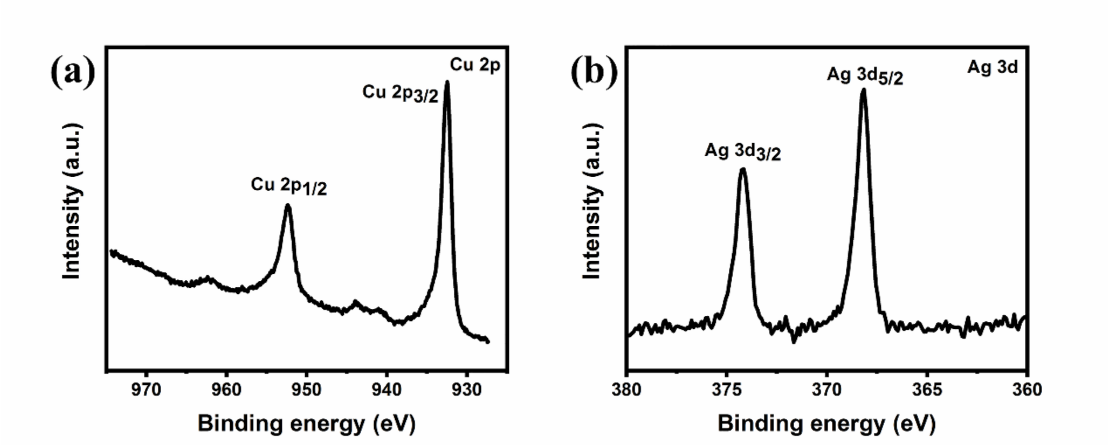

Figure S9. XPS spectra for (**a**) Cu and (**b**) Ag of Cu_2O-Ag nanocubes after CO_2RR.

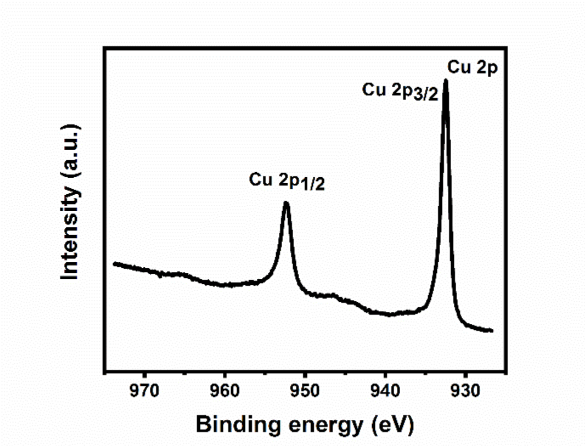

Figure S10. XPS spectra for Cu of Cu_2O nanocubes after CO_2RR.

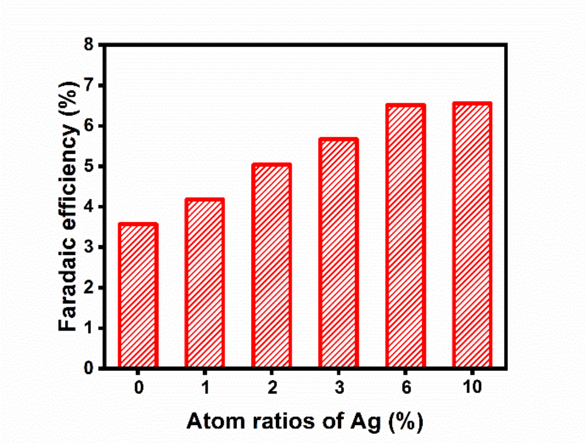

Figure S11. Faradaic efficiency of acetate of the different mass ratios of Ag in the Cu_2O-Ag nanocubes.

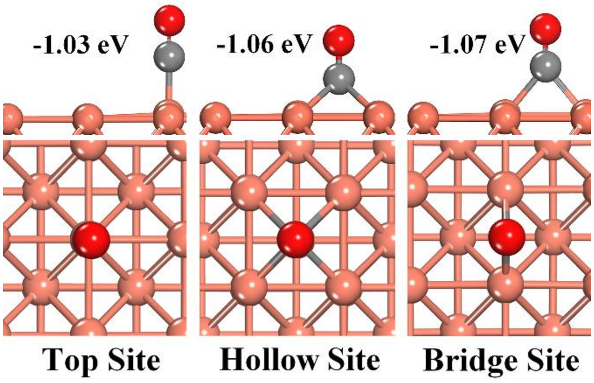

Figure S12. Adsorption energy and corresponding configuration of CO at three type sites on Cu(100) surface.

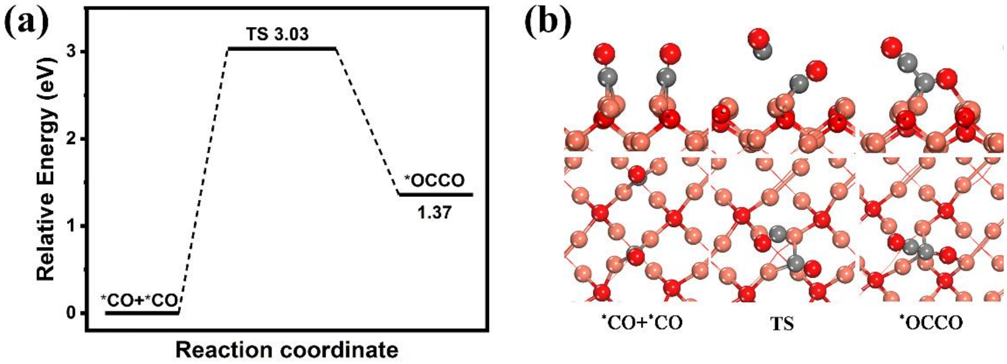

Figure S13. (**a**) Reaction energy barrier diagram of the C-C coupling step on the $Cu_2O(100)$ surface with the (**b**)corresponding configurations of two *CO forming an *OCCO. Light red, copper; grey, carbon; red, oxygen; TS, transition state.
